# Multiple Brown Tumors in Primary Hyperparathyroidism Causing Pathological Fracture: A Case Report of a 21-Year-Old Adult Male

**DOI:** 10.7759/cureus.35979

**Published:** 2023-03-10

**Authors:** Sultan Aldosari, Elham A Alghamdi, Ahmed Alragea

**Affiliations:** 1 Orthopedic Surgery, King Khaled University Hospital, King Saud Medical City, Riyadh, SAU; 2 Orthopedics, King Saud Medical City, Riyadh, SAU; 3 Orthopedic Surgery, King Saud University Medical City, Riyadh, SAU

**Keywords:** pathological fracture, osteitis fibrosa cystica, long bone fractures, primary hyperparathyroidism, parathyroid gland adenoma, brown tumor

## Abstract

Multiple brown tumors are more common in females and older age groups and an unlikely site is the long bones. We report a case of a 21-year-old male presenting with a pathological fracture at the left neck of the femur. Laboratory investigations showed elevated parathyroid hormone (PTH) and serum calcium levels (PTH-dependent hypercalcemia). A CT scan revealed multiple osteolytic lesions in the pelvis and femurs, and a Tc-99m sestamibi scan showed a solitary parathyroid adenoma. We demonstrate this rare case and illustrate the importance of the consideration of multiple brown tumors in young males presenting with multiple osteolytic lesions at the long bones in the differential diagnosis. Every physician needs to have a high clinical suspicion of primary hyperparathyroidism innovation, in those who present with osteolytic lesions, with respect to the patient’s age and gender.

## Introduction

Parathyroid glands secrete parathyroid hormone (PTH) under the control of the ionized calcium level [1]. Primary hyperparathyroidism is defined by increased PTH production caused by parathyroid adenoma in most cases (85%), followed by parathyroid hyperplasia (15%) and parathyroid carcinoma (<1%) [2,3]. Hyperparathyroidism causes bone metabolic disorders characterized by increased bone resorption and an increased frequency of bone remodeling, leading to increased bone porosity [3,4]. Primary hyperparathyroidism primarily targets the bones [5]. Common skeletal changes detected on plain radiographs in a patient with primary hyperparathyroidism include multiple osteolytic lesions, subperiosteal resorption, and osteopenia [5,6]. Due to advances in medical care, early diagnosis and treatment of hyperparathyroidism detected by routine laboratory findings have been increasing, so diagnosing primary hyperparathyroidism in the setting of multiple brown tumors is a particularly rare disorder today, except in cases of severe untreated primary hyperparathyroidism or parathyroid carcinoma associated with it [5].

Brown tumor, also called osteitis fibrosa cystica, is a benign intraosseous tumor that occurs due to osteoclast hyperactivity in hyperparathyroidism [7]. It is associated more commonly with primary hyperparathyroidism [8]. Approximately 3% of patients with primary hyperparathyroidism develop brown tumors [9]. The solitary adenoma is the most common cause of primary hyperparathyroidism, resulting in excessive parathyroid hormone secretion (PTH) [2,3]. It can involve any portion of the skeleton, including the head bones (commonly the mandible), pelvic bones, long bones, and ribs [3,4]. Brown tumors usually occur as solitary lesions but rarely present as multiple lesions, and they are usually reported in the case of reports [2,7,10].

The clinical presentation is variable, but some patients have nonspecific generalized muscle weakness, fatigue, recurrent urinary stones, and fractures [11]. Brown tumors are often misdiagnosed as primary skeletal neoplasms because of these clinical findings. Few cases of brown tumors with musculoskeletal manifestations and mimicking neoplasm lesions have been reported in the medical literature [11-13].

Plain radiographic findings include multiple osteolytic lesions, subperiosteal resorption, and osteopenia [6]. Sestamibi scanning is now the best modality for identifying adenomas. It has a reported sensitivity of 54% to 100% [14]. Mirels’ classification is a system for scoring impending pathologic fractures based on four characteristics: site, size, nature of the lesion, and presence of pain. According to Mirels’ recommendation, a score of 9 or greater determines prophylactic fixation. A score of 7 or less can be managed using radiotherapy and drugs [15].

The key treatment for primary hyperparathyroidism when imaging techniques show a parathyroid adenoma is the excision of enlarged parathyroid glands. Bone deformities caused by it usually spontaneously disappear with the treatment of hyperparathyroidism [14]. Postoperative complications include hungry bone syndrome, a serious complication, rapid and prolonged hypocalcemia, hypophosphatemia, and hypomagnesemia [16].

Brown tumors are more common in females than males, with a reported ratio of five females to four males; they also affect older patients, usually in the fifth and sixth decades of life [13,17].

Here, we describe a case of multiple brown tumors caused by a parathyroid adenoma that presented with a pathological fracture and was treated with surgical intervention.

## Case presentation

**Figure 1:**
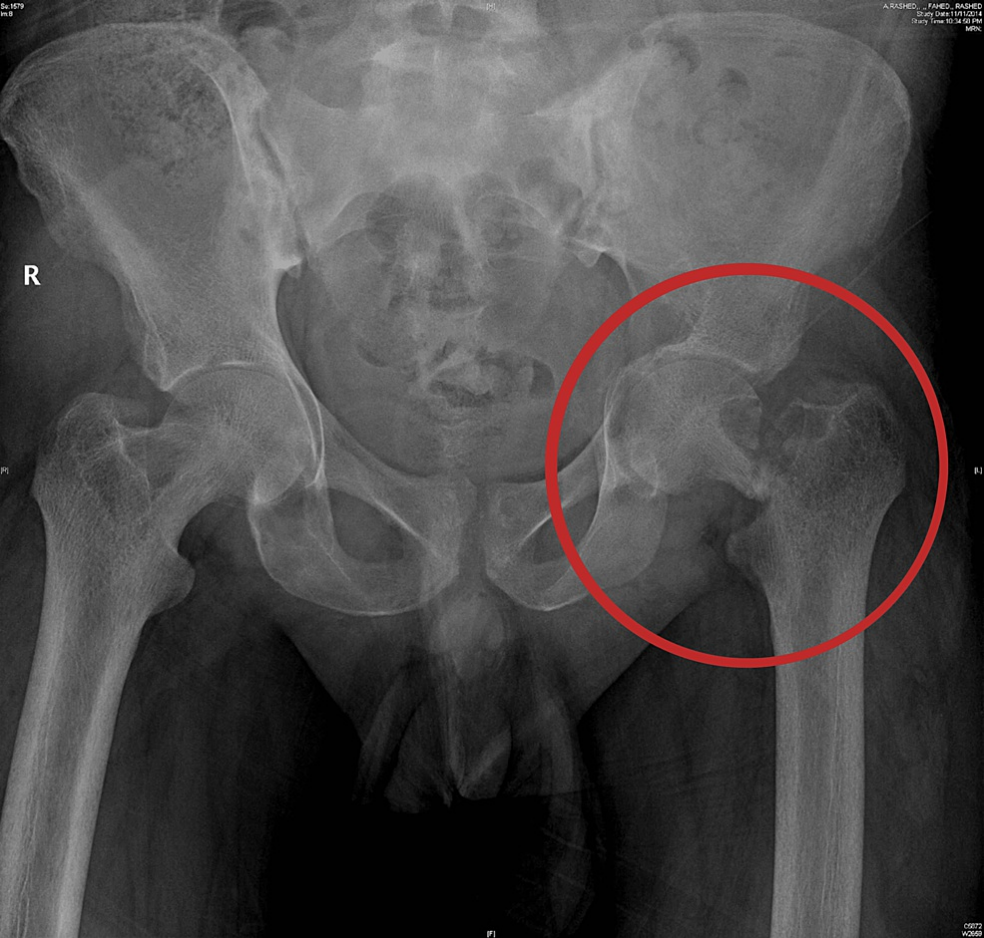
Plain radiograph of the pelvis: left neck of femur fracture, left protrusio acetabuli, and osteolytic lesion in the right neck of femur region

A 21-year-old adult male, with no relevant past medical and surgical history, presented to the emergency department complaining of severe left hip pain and an inability to bear weight after he slipped and fell from a 1-meter height. Immediately, he was unable to mobilize his left lower leg. He also complained of generalized body aches and easy fatigability. On examination, there was left hip joint tenderness and an external rotation deformity of the left lower leg. There were no wounds, and distal neurovascular examinations were intact. Examination of other joints was within normal limits. Pelvis (anteroposterior (AP)) and left hip (AP, lateral (LAT)) Plain radiographs were taken, and they showed a left neck femur fracture with an osteolytic lesion at the fracture line. There were multiple osteolytic lesions seen in the left acetabulum and right hip. Additionally, there was generalized osteopenia and protrusio acetabuli on the left side (Figure 1).

**Figure 2:**
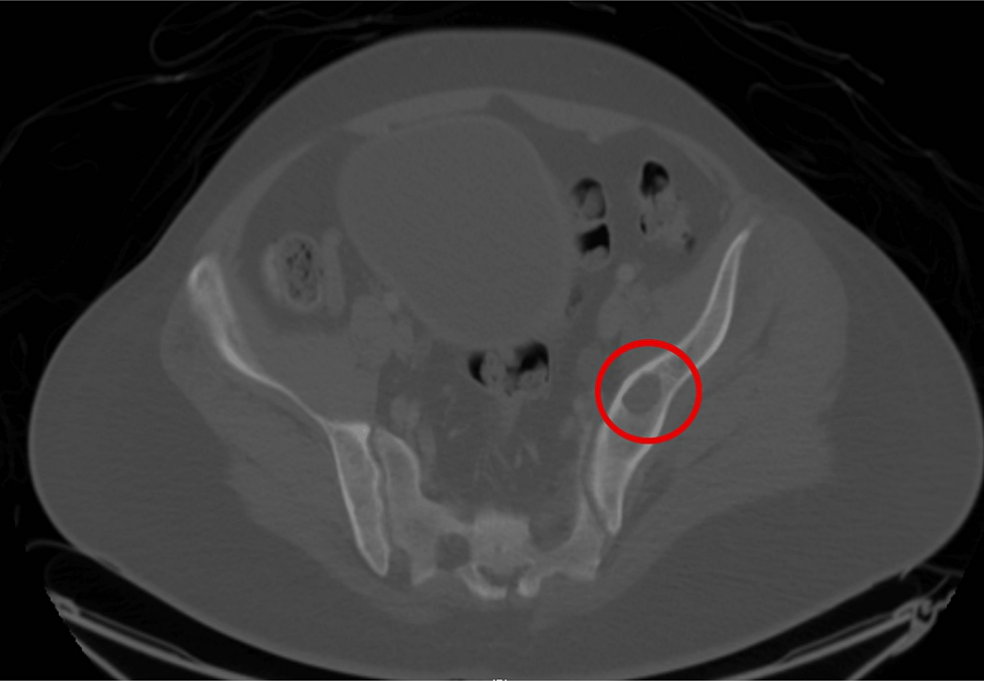
An axial CT scan of the pelvis revealed multiple osteolytic lesions and an acetabular protrusion

**Figure 3:**
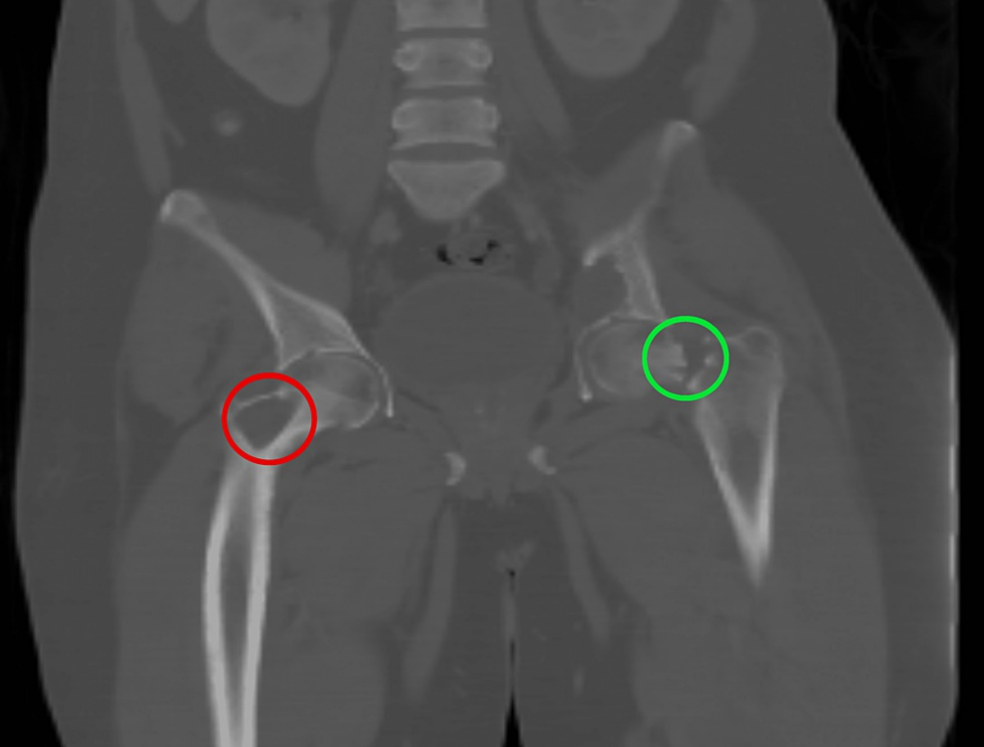
CT scan of the pelvis: coronal view, with red circle showing multiple osteolytic lesions and protrusion acetabuli and green circle showing a left neck of femur fracture

Laboratory examination showed a total serum calcium of 3.14 mm/L, corrected calcium of 3.3 mm/L, and low serum phosphorus and 25-hydroxyvitamin D levels with a high parathyroid hormone level of 107.5 pmol/liter. A CT scan showed multiple well-defined osteolytic lesions in the acetabulum and right femur neck (Figures 2-3).

**Figure 4:**
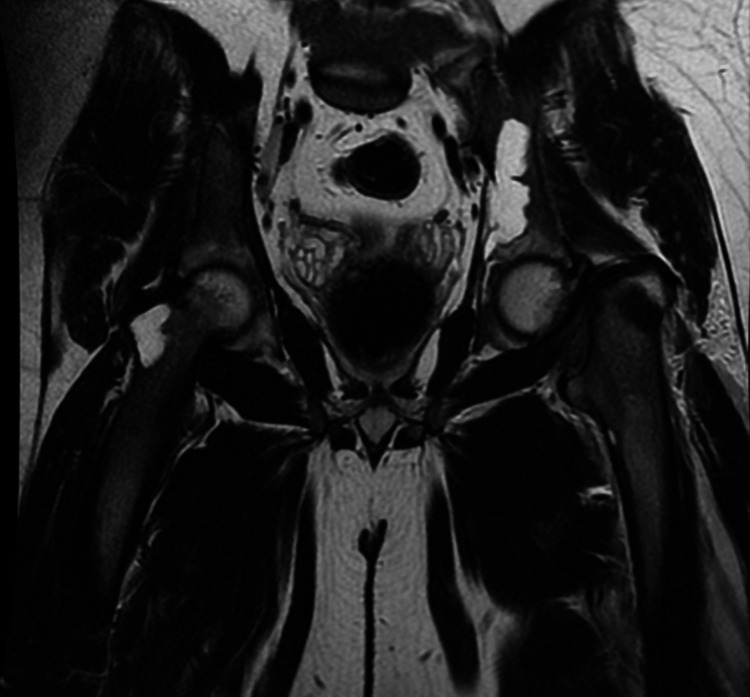
MRI hip: coronal T2 view showing multiple cystic lesions in the left acetabulum and right neck of femur region

MRI of the hips revealed multiple cystic lesions around the acetabulum that were hyperintense on T2-weighted images (Figure 4).

**Figure 5:**
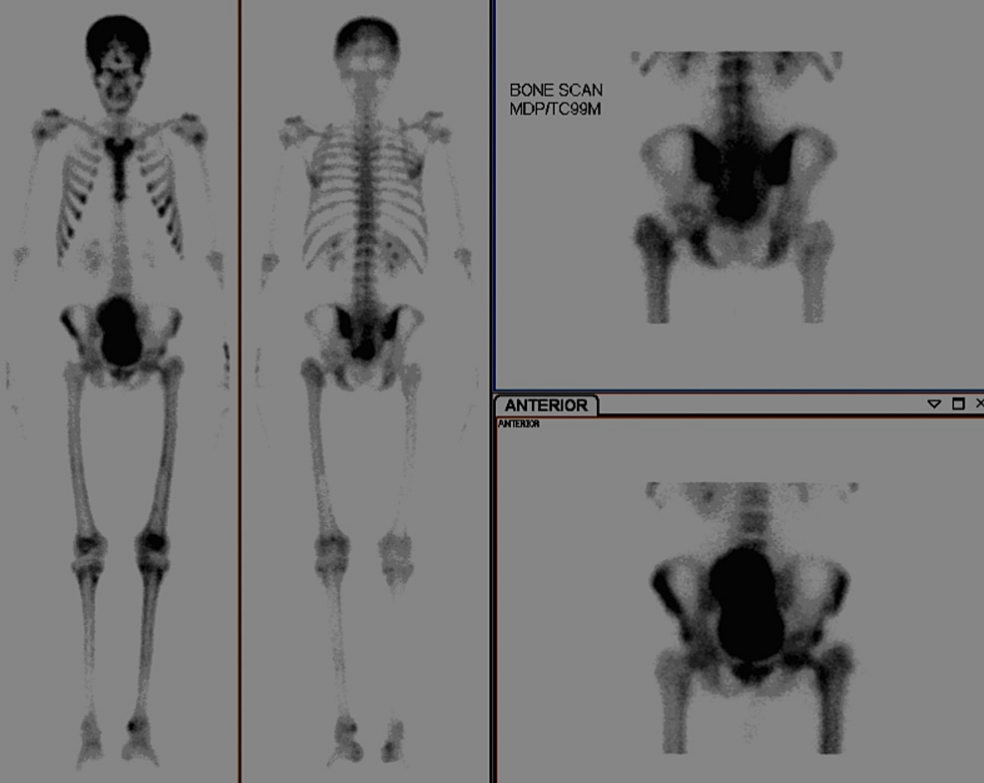
Bone scan showing increased uptake in the pelvic region, femur, ribs, and tibia

A bone scan showed increased abnormal focal uptake at the femur, tibia, and ribs (Figure 5).

**Figure 6:**
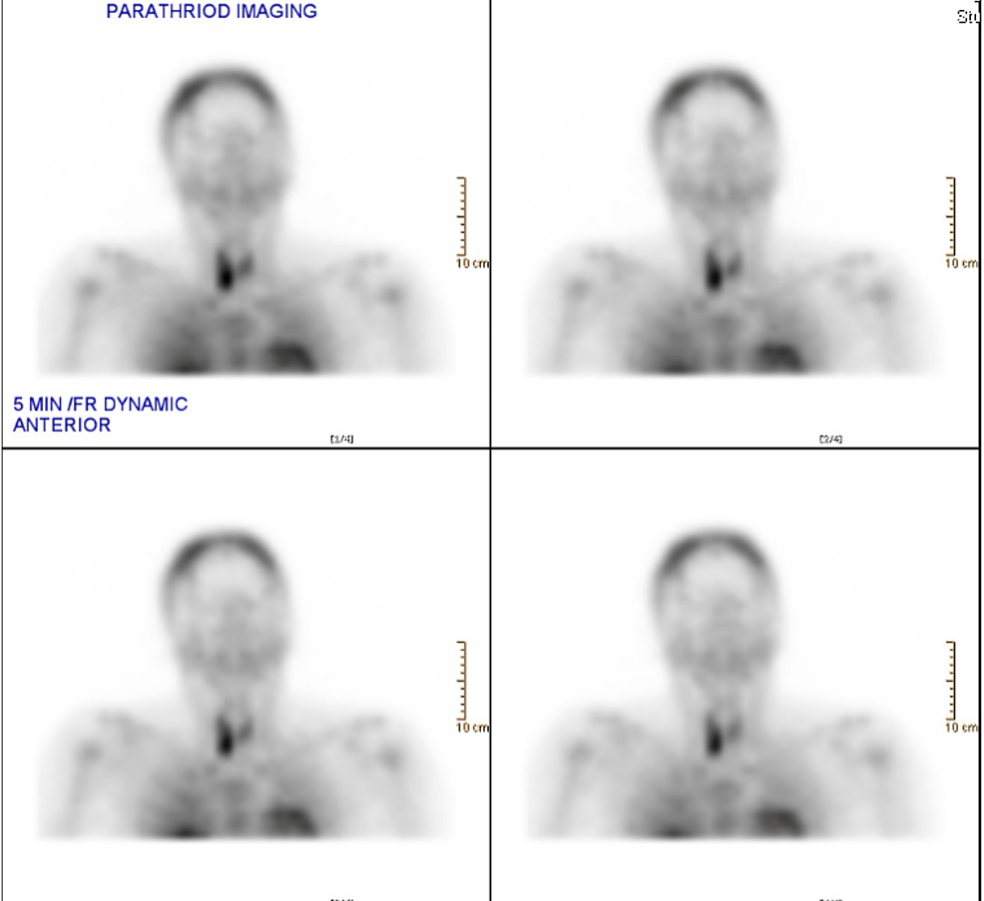
Sestamibi scan showed increased uptake at the right parathyroid

Technetium-99m sestamibi images showed increased uptake by the right parathyroid gland (Figure 6).

Dual-energy X-ray absorptiometry (DXA) evaluation showed the presence of osteoporosis.

The diagnosis of primary hyperparathyroidism with a right parathyroid adenoma was established. After determining that the patient had osteolysis, intravenous fluids, a loop diuretic, and vitamin D3 were administered to the patient.

Blood investigations (a bone profile and renal function test) were taken daily to monitor corrected calcium levels. On the third day of admission, the correct calcium level was average, 2.3 mm/l. Right parathyroidectomy was done to treat the primary cause, and the parathyroid hormone level dropped by more than 50% immediately post-excision. The histopathology laboratory reported no malignant cells to be found. On day one postoperatively, the patient developed “hungry bone” syndrome, with the crisis manifesting as muscle spasms, numbness, and tingling in the hands, feet, and face, requiring calcium and vitamin D treatment. The patient was treated accordingly.

**Figure 7:**
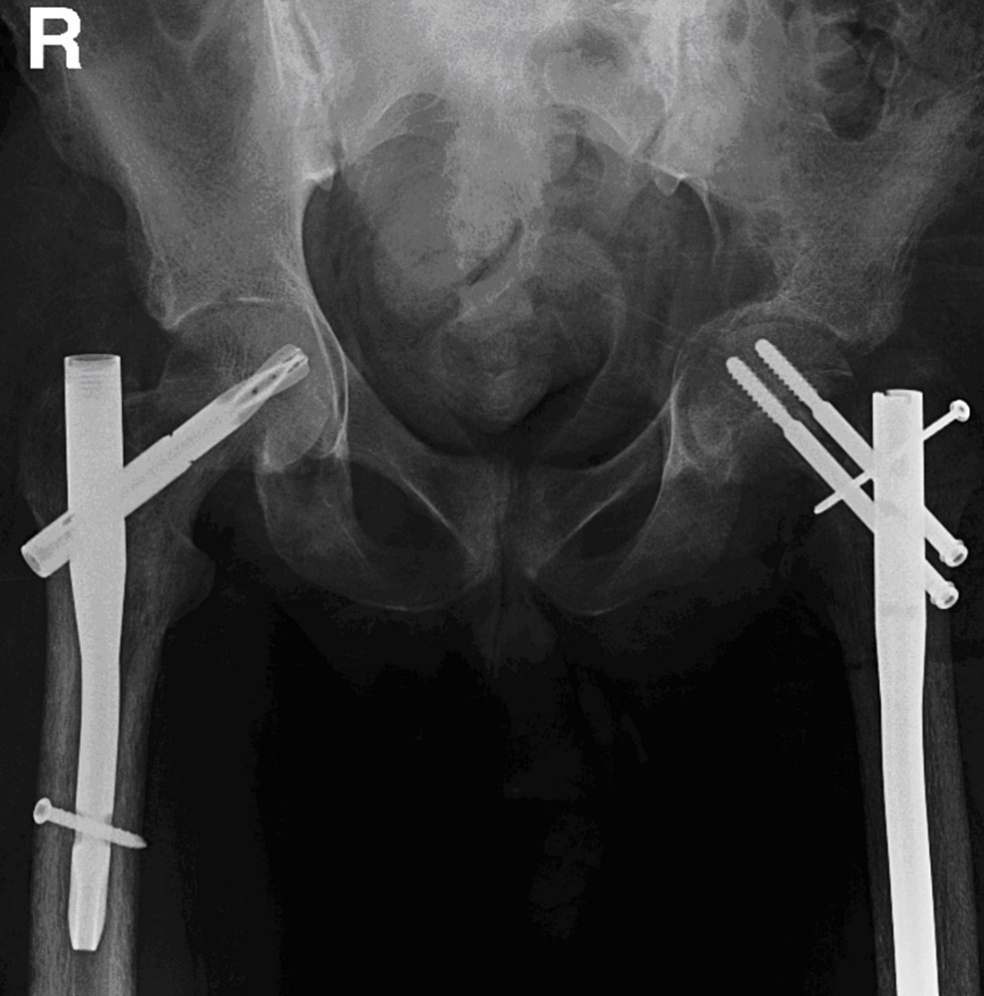
Plain radiograph of the pelvis: post left cephalomedullary nail and right proximal femoral nail insertion

Day three postoperatively normalized his parathyroid hormone and corrected his calcium level. Then, the patient was taken to the operation room by an orthopedic surgeon for a bone biopsy from the distal left femur and sent to a histopathology laboratory for a frozen section diagnosis. It came as bone marrow elements with focal hemosiderin deposits and fibrosis, consistent with a diagnosis of the brown tumor. Due to the patient Mirels’ score of 10, the operation proceeded to close reduction and cephalomedullary nail (reconstruction nail) for the left neck of femur fracture and prophylactic fixation for the right hip by proximal femoral nail (PFNA) (Figure 7).

Postoperatively, the patient started to mobilize non-weight-bearing (wheelchair). The patient was discharged on oral calcium, a vitamin D supplement, and regular follow-up in the outpatient department with the endocrine clinic and orthopedic clinic.

## Discussion

Brown tumors of the bones are a late manifestation of severe hyperparathyroidism and primarily affect the cortical bones. Microhemorrhages and microfractures characterize it, leading blood products like hemosiderin to deposit and become reddish-brown, hence the tumor name [3,6]. The common sites of brown tumors are the bones of the hands, jaw, skull, pelvis, clavicle, ribs, femur, and spine [3,4]. Our patient had a femur fracture with an osteolytic lesion at the fracture line. There were multiple osteolytic lesions seen in the left acetabulum and right hip. Additionally, there was generalized osteopenia and protrusion acetabuli on the left side. A complete blood test helps differentiate the cause of this fracture and osteolytic lesion. Multiple osteolytic lesions should draw significant attention from every physician. They were taking into consideration metastatic bone tumors, multiple myeloma, or brown tumors in the differential. A complete tumor survey, including a bone scan, chest and abdominal imaging, and relevant laboratory studies, including cell counts, biochemical analyses, and tumor marker levels, might help diagnose [7]. In our case, laboratory and imaging evidence indicated that brown tumors are highly possible. Screening for familial hyperparathyroidism is done because of the young onset of hyperparathyroidism. Multiple endocrine adenomas (MEN) were ruled out by confirming the absence of pituitary, and gastro-entero-pancreatic (GEP) neuroendocrine tumors (NETs). A bone biopsy from the distal left femur was taken, and a diagnosis consistent with the diagnosis of the brown tumor was made.

Brown tumors are more common in females, with a female-to-male ratio of 5:434200405; they are also more common in older age groups, usually in the 50s and 60s [13,17]. Multiple brown tumors in males have been reported in very few cases, rarely affecting the long bones, and reported in few cases [5,13,17]. Panagopoulos et al. reported the case of a 53-year-old male who presented with multiple osteolytic lesions of the distal ulna and radius. Their patient was initially misdiagnosed with giant cell tumors of the distal ulna and radius and underwent unnecessary surgical intervention; later on, multiple brown tumors due to primary hyperparathyroidism caused by parathyroid gland carcinoma were correctly identified [5]. Bal, Jaspriya, et al. reported the case of a 33-year-old male who presented with a lytic lesion of the right tibia, and a diagnosis of multiple brown tumors due to a parathyroid adenoma was made [13]. Luz M. Morán et al. reported a 45-year-old male who presented with multiple osteolytic lesions in the femurs and right pubis, and a definitive diagnosis of brown tumors caused by hyperparathyroidism due to a parathyroid adenoma was made [17]. In all three cases reported in the literature, although the presentation and site of the lesions were similar, the patients’ ages were older than the 30s. In our case, our patient was a 21-year-old male, who was younger than the affected males in the literature. To make a long story short, we conferred on the case of a young man with multiple brown tumors associated with primary hyperparathyroidism, which presented as a left neck femur fracture.

## Conclusions

This article is a case report explaining the occurrence of a brown tumor in a young male with primary hyperparathyroidism. A brown tumor is a benign intraosseous tumor caused by osteoclast hyperactivity. It is a rare tumor with an incidence of 3%. It is more common in females and older age groups and uncommon in long bones. This case demonstrates a young male patient who presented with a pathological fracture in the femur and was initially diagnosed with hyperparathyroidism due to the presence of an adenoma. Moreover, on further investigations after parathyroidectomy, we found multiple brown tumors with multiple osteolytic lesions and increased levels of calcium and PTH. We performed a right parathyroidectomy, a closed reduction, a cephalomedullary nail (reconstruction nail) for the left neck of the femur fracture, and prophylactic fixation for the right hip with a proximal femoral nail to our patient. He will receive regular follow-ups to track the disease’s further development. Finally, every physician needs to have a high clinical suspicion of primary hyperparathyroidism innovation, who presented with osteolytic lesions, with respect to the patient’s age and gender.
